# Embolic Pneumonectomy for the Treatment of Proximal Pulmonary Artery Pseudoaneurysm

**DOI:** 10.7759/cureus.26487

**Published:** 2022-07-01

**Authors:** Christopher J Sommer, Daniel O’Neal, R. Hampton Andrews

**Affiliations:** 1 Department of Radiology, Eastern Virginia Medical School, Norfolk, USA

**Keywords:** pulmonary artery pseudoaneurysm, computed tomography pulmonary angiography, massive hemoptysis, interventional radiology-guided embolization, transarterial coil embolization

## Abstract

Pulmonary artery pseudoaneurysm is an uncommon and potentially fatal abnormality. It has been described from a wide variety of etiologies, including infectious, iatrogenic, neoplastic, congenital, and traumatic causes. There are currently no published consensus guidelines for the diagnostic testing and management of pulmonary artery pseudoaneurysm. This case report presents an uncommon case of pulmonary artery pseudoaneurysm emerging from a non-small cell lung cancer that was successfully managed using coil embolization.

## Introduction

Pulmonary artery pseudoaneurysm is a rare finding that requires a timely and accurate diagnosis. Pseudoaneurysms are caused by a disruption in only the adventitial layer of the artery, giving these defects a particularly high risk of rupture and subsequent hemorrhage. The most common cause of pulmonary pseudoaneurysms is infection (Rasmussen’s aneurysm, necrotizing pneumonia, and infectious endocarditis); however, a multitude of additional etiologies have been described. Cases have been described arising from pulmonary embolism, neoplasia, intravascular or extravascular trauma, pulmonary hypertension, vasculitides, and Marfan’s syndrome [1-4].

The presenting symptoms of pulmonary artery pseudoaneurysm vary widely but most commonly include hemoptysis, shortness of breath, chest pain, and cough. A subsection of patients present with no pulmonary symptoms at all. Diagnosis is often found incidentally on imaging, but in patients with concerns for life-threatening hemoptysis, computed tomography pulmonary angiography (CTPA) alongside bronchoscopy is currently the most widely supported diagnostic approach in the literature [2].

The most concerning complications emerging from pulmonary artery pseudoaneurysms are rupture and massive hemoptysis. Mortality rates from massive hemoptysis have been estimated from 9% to 50%. Since life-threatening hemoptysis can occur at thresholds as low as 100 mL of expectorated blood over a 24-hour period, it is of utmost importance that stasis is quickly and effectively achieved [4-6]. With most cases of massive hemoptysis arising from the bronchial arteries, hemorrhage from the pulmonary artery is a far less common source accounting for between 5% and 11% of cases of life-threatening hemoptysis [3,4,7]. Hemoptysis arising from such pulmonary artery pseudoaneurysms can be treated with surgical resection, bronchoscopy, or endovascular coil embolization [2,5]. The limited literature available suggests that endovascular coil embolization is a reasonable and safe management approach for these lesions [3,4,8,9].

## Case presentation

**Figure 1:**
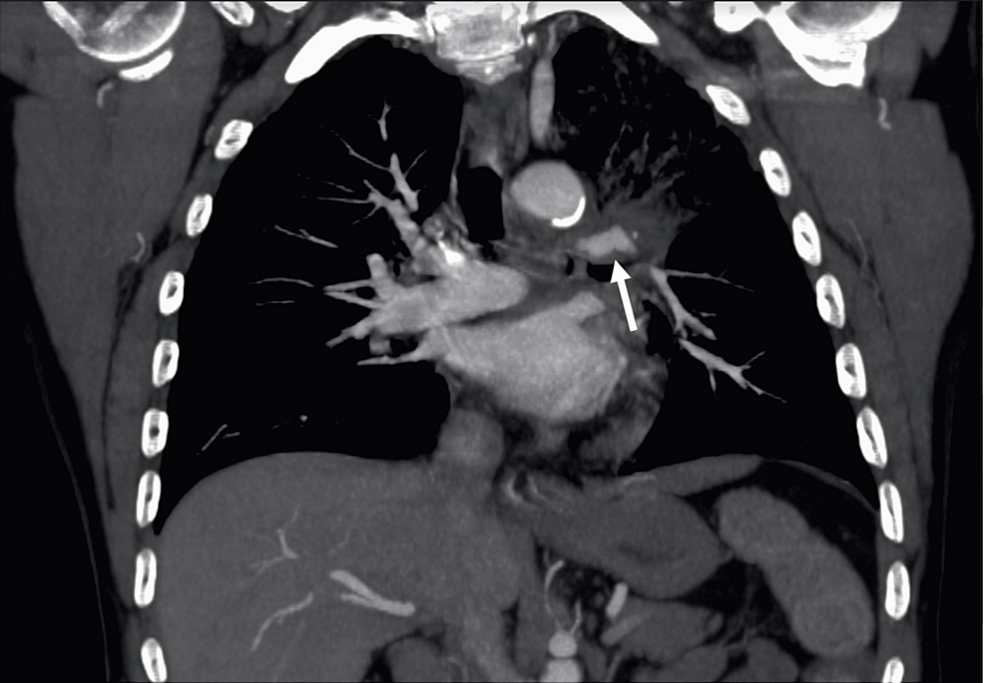
Coronal CT pulmonary angiogram showing the left suprahilar mass surrounding the left pulmonary artery with an associated pseudoaneurysm (arrow).

**Figure 2:**
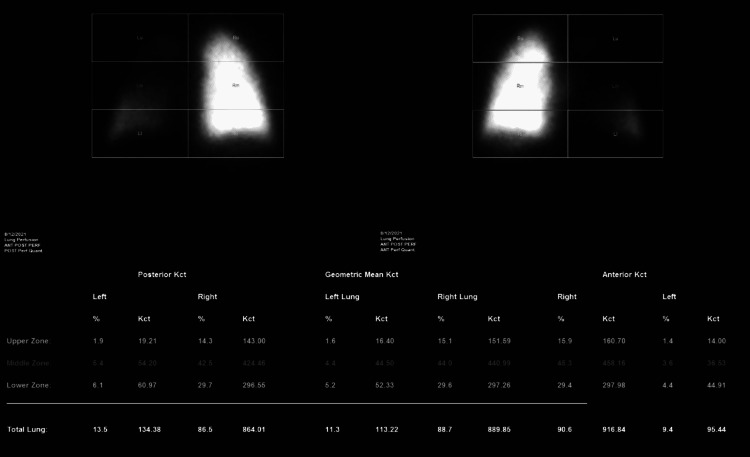
Perfusion scan demonstrating significant hypoperfusion of the left lung.

A 58-year-old male presented to the emergency department at our institution for 24 hours of hemoptysis. The patient had a prior history of chronic obstructive pulmonary disease, recently diagnosed stage III non-small cell lung cancer, and status post radiation and chemotherapy. His medical history was also remarkable for a long history of smoking and alcohol abuse. The patient’s hemoptysis began spontaneously, and he was in acute distress upon arrival. On physical examination, he was found to be tachycardic and hypotensive with bilateral rales on auscultation. Computed tomography pulmonary angiography (CTPA) was obtained and demonstrated enlarging left suprahilar mass with associated fusiform bulging of the adjacent left pulmonary artery, as well as consolidation and ground-glass opacities throughout the left upper lobe (Figure 1). This had progressed from a prior CTPA taken 40 days earlier and was interpreted as worsening malignancy with a new left pulmonary artery pseudoaneurysm and likely pulmonary hemorrhage given the recent history of hemoptysis. The patient was admitted to the intensive care unit, where an endotracheal tube was placed, and a bronchoscopy was performed, demonstrating an erosive tumor within the left mainstem bronchus. Subsequent nuclear medicine perfusion scanning showed significant left lung hypoperfusion (Figure 2). The decision was then made to perform diagnostic pulmonary artery angiography in the angiography suite with possible embolization of the bleeding left pulmonary artery.

**Figure 3:**
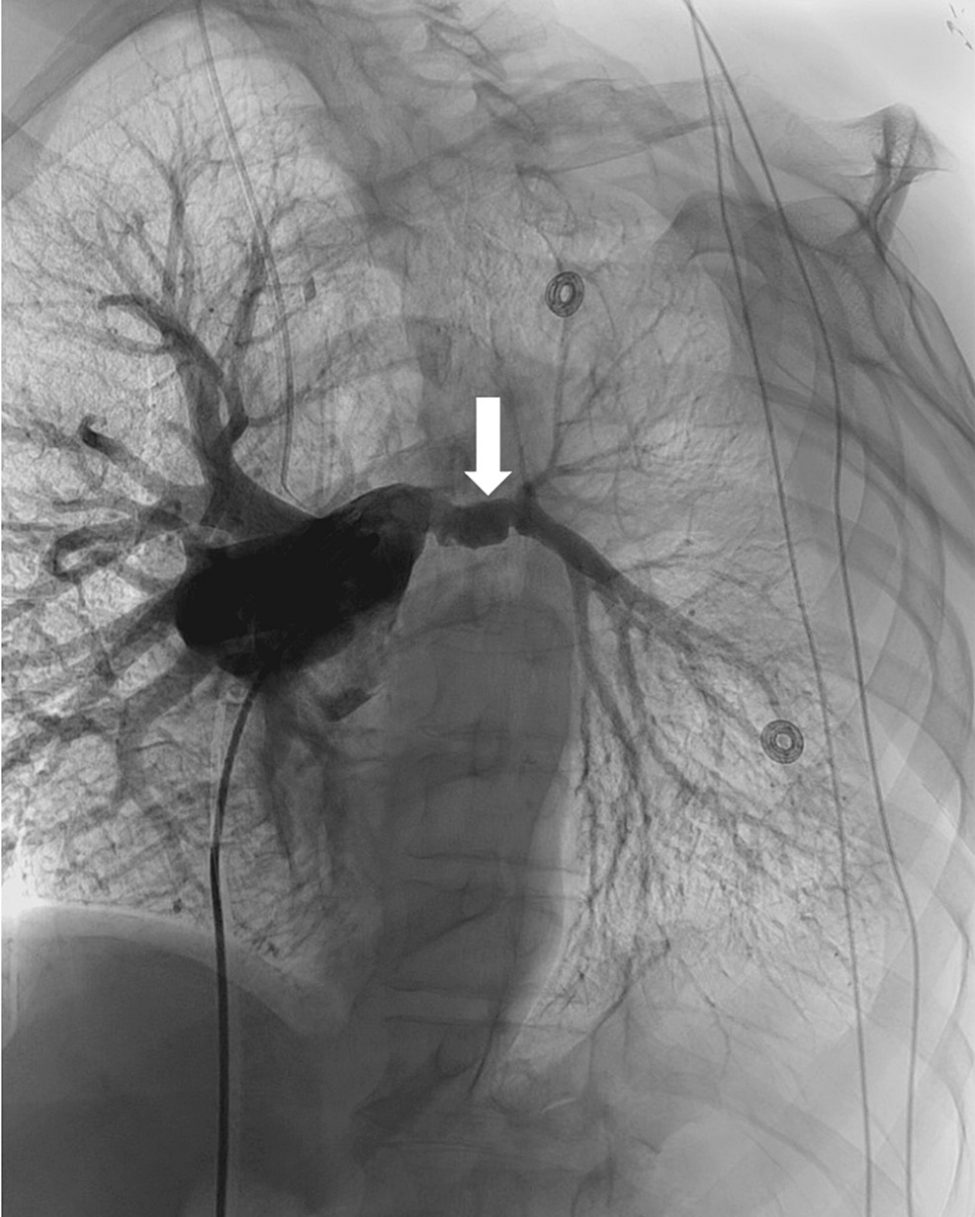
Main pulmonary artery angiography showing the pseudoaneurysm at the proximal left pulmonary artery (arrow).

**Figure 4:**
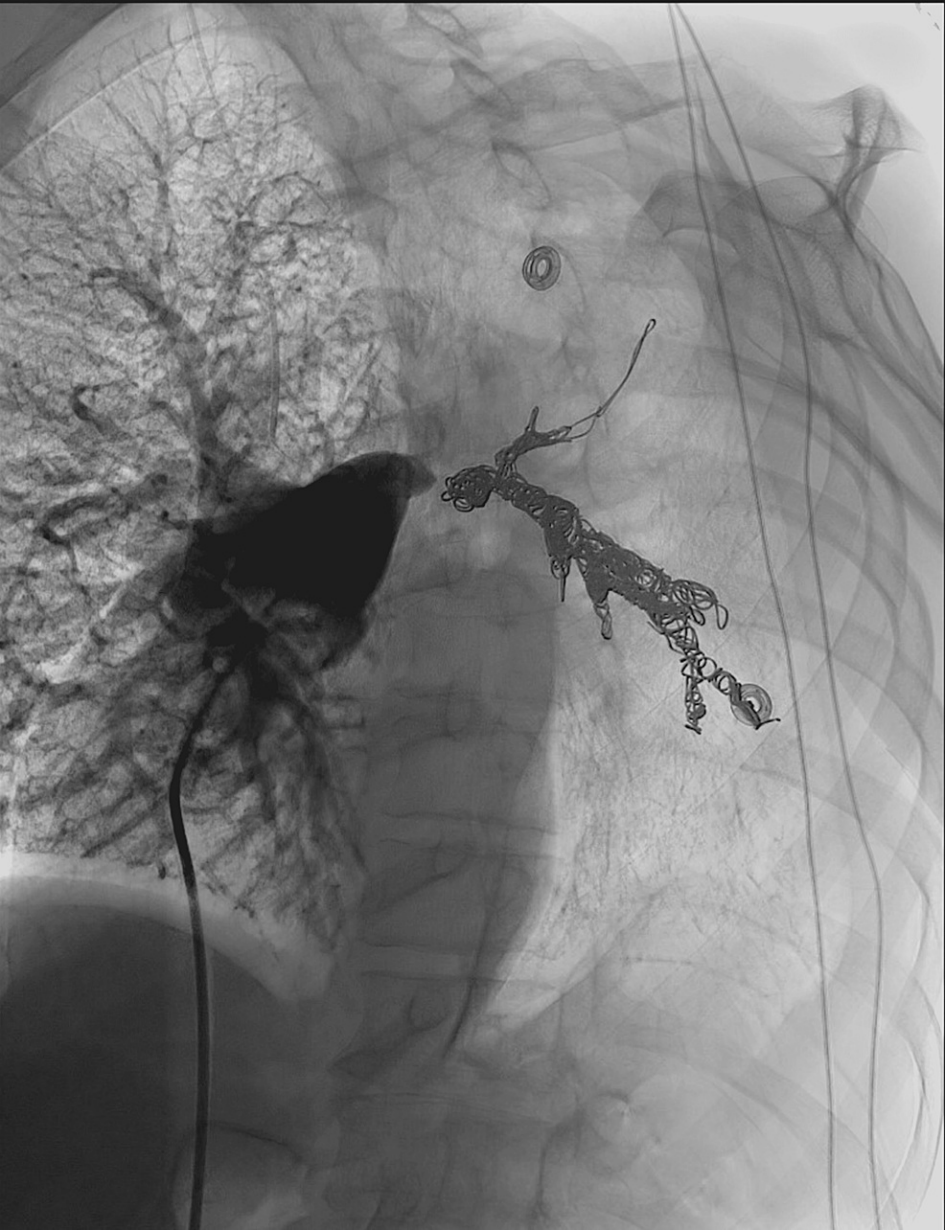
Main pulmonary artery angiography showing successful coil embolization of the left pulmonary artery. No contrast is seen past the coil mass, and the left pulmonary artery pseudoaneurysm is no longer visualized.

The right common femoral vein was accessed, and a 7F vascular sheath was placed. A 6F angled pigtail catheter was then advanced into the IVC, through the right heart, and into the main pulmonary artery. Diagnostic flush angiography of the pulmonary arterial tree was performed at 40° left anterior oblique, which demonstrated an irregular proximal left main pulmonary artery and normal right pulmonary artery (Figure 3). This ideal imaging angle was predetermined using three-dimensional software to manipulate the CTPA images. The pigtail catheter was exchanged for a 5F Berenstein catheter over a wire and was used to select the left main pulmonary artery. Diagnostic angiography of the left main pulmonary artery was performed and showed irregular caliber and arterial wall contour with a pseudoaneurysmal outpouching proximally. A 2.8F Progreat microcatheter and microwire system was then advanced through the catheter and across the pseudoaneurysm into the distal left pulmonary arterial tree. Diagnostic angiography was performed, which demonstrated normal branching of the segmental arteries and allowed the evaluation of catheter positioning for embolization. The 5F catheter was then advanced over the microcatheter into a distal segmental branch of the left lower lobe pulmonary artery. The microcatheter was removed, and nester pushable coils were passed through the 5F catheter, embolizing the left lower segmental branches of the left pulmonary artery. The 2.8F Progreat microcatheter was then reinserted and positioned within the left pulmonary artery, proximal to the nester coils, and coil embolization was further performed using Penumbra Ruby coils, Penumbra Packing coils, and Penumbra pod coils under fluoroscopic guidance. A final angiogram demonstrated no active extravasation and satisfactory hemostasis with no antegrade blood flow distal to the coil mass (Figure 4). There were no immediate complications following the procedure, and the patient was transferred to recovery in stable condition.

## Discussion

This is a case of life-threatening hemoptysis from a pulmonary artery pseudoaneurysm secondary to a non-small cell lung carcinoma. The limited literature available suggests that this is a relatively unusual cause of pulmonary artery pseudoaneurysm, accounting for just 12.5% of those documented [4]. Prior to intervention, the diagnostic standard for massive hemoptysis, CT angiography alongside bronchoscopy, was performed. Often, hemoptysis can be treated with bronchoscopic intervention; however, this was not considered an ideal option for this patient due to the size of the lesion. This was when the interventional radiology department was contacted to see if there was an intervention they could offer.

Although there is limited literature on the treatment of pulmonary artery pseudoaneurysms endovascularly, we felt that coil embolization would be ideal. Endovascular treatment of vascular abnormalities has become increasingly popular since the 1970s, offering a less invasive alternative to surgical management with reduced morbidity and mortality. We felt that a sac packing technique with additional embolization of both the efferent and afferent portions of the affected vessel would be appropriate for achieving hemostasis in this case. This technique involves the placement of malleable metal coils into the vascular sac until stasis is achieved. The placement of these coils functions to decrease blood flow, produce thrombosis, and encourage inflammation within the targeted space [10,11]. Our biggest barrier to attempting coil embolization was whether the patient could tolerate losing blood supply to his left lung as the pseudoaneurysm was located at the main left pulmonary artery. To gauge this, we decided to perform a nuclear medicine pulmonary perfusion study. This can be used to calculate the percentage of pulmonary blood supply that the affected left lung represented. When the results showed that there was already significant hypoperfusion of the left lung due to the mass, we felt comfortable proceeding with coil embolization.

Coil embolization was then performed starting from the unaffected segmental left lower lobe pulmonary arteries back across the pseudoaneurysm to the proximal left main pulmonary artery. This allowed for stable coiling while also treating other left pulmonary vessels involved with the mass. Subsequent postembolization imaging showed effective stasis. This procedure allowed the patient to be bridged to palliative care, and the patient was able to be discharged home. Given the relative sparsity of information on treating pulmonary artery pseudoaneurysms, hopefully, this case can provide a framework for future cases and improve patient outcomes.

## Conclusions

Pulmonary artery pseudoaneurysm is a rare finding with limited literature discussing the treatment of this condition. The high rate of mortality with rupture requires prompt recognition by all clinicians so treatment can occur prior to or in the setting of hemorrhage. Cases like this are needed for the further education of clinicians because there are no uniform guidelines for the treatment of pulmonary artery pseudoaneurysms. In our particular situation, we found coil embolization to be an effective solution while further guidelines are being developed for the treatment of pulmonary artery pseudoaneurysm.
